# Brd4-Mediated Nuclear Retention of the Papillomavirus E2 Protein Contributes to Its Stabilization in Host Cells

**DOI:** 10.3390/v6010319

**Published:** 2014-01-20

**Authors:** Jing Li, Qing Li, Jason Diaz, Jianxin You

**Affiliations:** Department of Microbiology, University of Pennsylvania School of Medicine, Philadelphia, PA 19104, USA; E-Mails: jing8@mail.med.upenn.edu (J.L.); sunnymaylq@hotmail.com (Q.L.); jdia@mail.med.upenn.edu (J.D.)

**Keywords:** papillomavirus, E2, Brd4, stability

## Abstract

Papillomavirus E2 is a multifunctional viral protein that regulates many aspects of the viral life cycle including viral episome maintenance, transcriptional activation, and repression. E2 is degraded by the ubiquitin-proteasome pathway. Cellular bromodomain protein Brd4 has been implicated in the stabilization of the E2 protein. E2 normally shuttles between the cytoplasm and the nucleus. In this study, we demonstrate that E2 ubiquitylation mostly occurs in the cytoplasm. We also find that the interaction with Brd4 promotes nuclear retention of papillomavirus E2 proteins and contributes to their stabilization in the nucleus. Compared to wild type E2 proteins, nuclear-localization-defective mutants are rapidly degraded by the ubiquitin-proteasome pathway; however, co-expression of Brd4 redirects these mutants into the nucleus and significantly increases their stability. We further demonstrate that tethering E2 proteins to chromatin as either double-bromodomain fusion proteins or histone 2B (H2B) fusion proteins significantly stabilizes the E2 proteins. Our studies suggest that chromatin recruitment of the E2 protein via interaction with Brd4 prevents E2 ubiquitylation and proteasomal degradation in the cytoplasm, leading to its stabilization in the nucleus. These studies bring new insights for understanding Brd4-mediated E2 stabilization, and provide an additional mechanism by which the chromatin-associated Brd4 regulates E2 functions.

## 1. Introduction

Papillomaviruses (PVs) are small DNA tumor viruses that induce benign and malignant epithelial lesions in the infected host [1]. Human papillomaviruses (HPVs) are classified into high-risk and low-risk types, depending on the potential for induction of malignant transformation. High-risk HPVs are strongly associated with cervical cancer [2]. PVs have a specific tropism for squamous epithelial cells and must infect cells within the dividing basal layer. The productive life cycle of HPV is intimately tied to the differentiation program of the host squamous epithelium. After infecting basal cells, the viral genomes are established as extrachromosomal elements (episomes). The various phases of the HPV life cycle are controlled through tightly regulated activation of the early and late viral promoters as the infected basal cell migrates towards the epithelial surface [3]. 

PV E2 is a regulatory protein essential for creating favorable cellular conditions for the establishment of infection and the proper completion of the viral life cycle. E2 consists of an N-terminal transcriptional activation domain (TAD) linked by a flexible hinge to a C-terminal DNA binding/dimerization domain [4]. E2 binds to a number of E2 binding sites in the long control region (LCR) of the PV genome in a cooperative manner to achieve a tight regulation of the viral early promoter during the differentiation-dependent stages of the viral life cycle. E2 plays important roles in viral episome maintenance in host cells; it also interacts with E1, another viral protein, to achieve viral genome replication [1]. 

E2 proteins from both HPVs and bovine papillomaviruses (BPVs) are short-lived in host cells. The stability of E2 is regulated by ubiquitin-proteasome-mediated protein degradation [5,6,7]. Both, E2 phosphorylation and the host cell sumoylation, have also been implicated in the regulation of E2 protein stability [5,7,8]. In addition, Cullin-3 and SCF^Skp2^ have been identified as major ubiquitin ligases involved in E2 ubiquitylation and degradation [9,10]. 

Our previous proteomic study identified the Brd4 protein as a major chromatin receptor for E2 proteins [11]. Brd4 is a member of the bromodomain and extra-terminal (BET) family of proteins, which harbor double bromodomains [12]. Brd4 binds to acetylated histone H3 and H4 through its N-terminal bromodomains and associates with both interphase chromatin and mitotic chromosomes [13]. E2 interacts with the C-terminal domain (CTD) of Brd4 [11]; this interaction has been implicated in E2’s multiple functions in the viral life cycle, including stable maintenance of viral genomes in dividing cells, viral transcriptional activation, and repression of viral oncogene expression from the integrated viral genome in HPV-positive cancer cells [11,14,15,16,17,18,19,20,21,22,23,24]. More recently, several studies have suggested that Brd4 plays a role in E2 stabilization [9,25,26]. It is established that Brd4 facilitates E2 binding to E2’s cognate sequences in HPV chromatin in part through enhancing E2 protein stability [25]. Proteasomal degradation of the papillomavirus E2 protein was found to be inhibited by overexpression of Brd4 [26]. Zheng *et al*. showed that expression of the Brd4 CTD blocks the interaction between E2 and the Cullin-3 ubiquitin protein ligase, contributing to E2 stabilization [9]. 

In this study, we find that E2 ubiquitylation occurs mostly in the cytoplasm. We demonstrate that binding of E2 to Brd4 prevents the nuclear export of both HPV16 and BPV1 E2 proteins and leads to their accumulation inside the nucleus. Compared to wild type E2 proteins, E2s defective in nuclear localization are highly unstable and rapidly degraded by the proteasome. However, co-expression of the Brd4 molecules recruits the nuclear-import-defective mutant into the nucleus and dramatically increases its stability. We also demonstrate that the HPV16 E2 mutant (R37A/I73A), which is defective in Brd4 binding, is less stable than wild type HPV16 E2. We then show that promoting E2 chromatin association by fusing them to either Brd4 double bromodomains (BDI/II) or histone H2B significantly enhances E2 stability. Taken together, our studies demonstrate that interaction with Brd4 contributes to E2 nuclear retention and prevents its degradation in the cytoplasm. These studies suggest that the Brd4-mediated E2 chromatin association and stabilization may represent a mechanism for PVs to evade the antiviral protein degradation present in the cytoplasm.

## 2. Results and Discussion

### 2.1. Results

#### 2.1.1. E2 Is Mainly Ubiquitylated in the Host Cytoplasm

**Figure 1:**
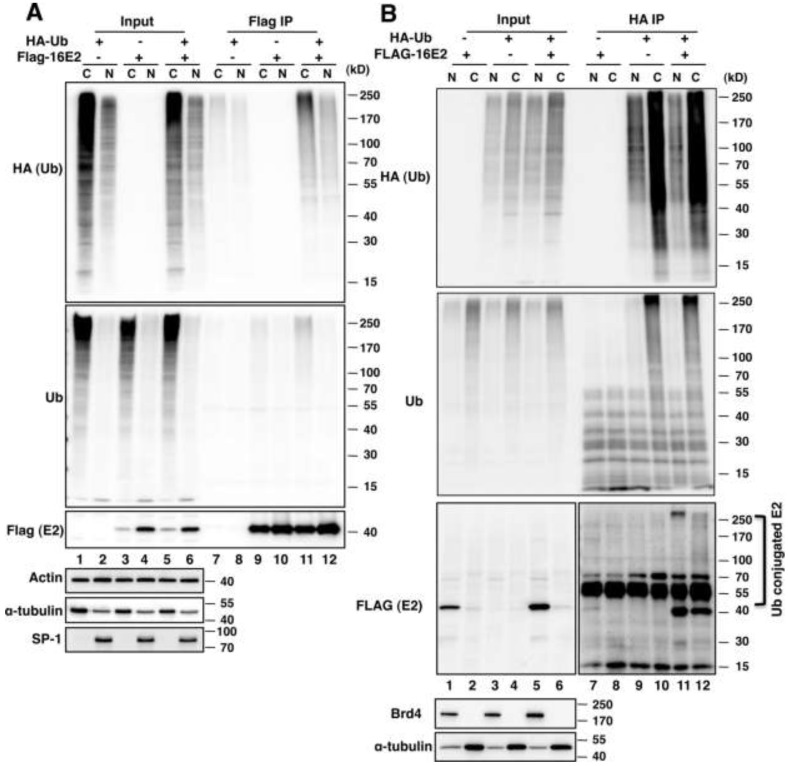
E2 is predominantly ubiquitylated in the host cytoplasm. HEK293T cells were transfected with plasmids encoding HA-tagged ubiquitin, FLAG-tagged HPV16 E2 or both as indicated. Forty-four hours post-transfection, the cells were treated with 30 µM MG132 for 4 h, and then lysed to obtain cytoplasmic extracts and nuclear extracts. (**A**) FLAG immunoprecipitation was performed to isolate ubiquitylated HPV16 E2 from both cytoplasmic extracts (C) and nuclear extracts (N); (**B**) HA immunoprecipitation was performed to isolate HA-ubiquitin conjugated proteins. The purified immune complexes were resolved on a sodium dodecyl sulfate-Polyacrylamide gel electrophoresis (SDS-PAGE) gel. Western blotting was performed using antibodies recognizing HA (for HA-tagged ubiquitin), Ub (for total ubiquitin), FLAG (for E2), actin, tubulin, Brd4 and SP-1. Tubulin was used as a cytoplasmic protein marker. Brd4 and SP-1 serve as nuclear markers.

E2 proteins are short-lived in host cells, but primarily function in the nucleus. While it is known that E2 degradation is mediated by the ubiquitin-proteasome pathway [5,6,7], where E2 is ubiquitylated and degraded has not yet been examined. To answer this question, we examined E2 ubiquitylation in both the cytoplasm and the nucleus. HEK293T cells were transfected with plasmids encoding HA-tagged ubiquitin, FLAG (DYKDDDDK)-tagged HPV16 E2, or both, as indicated in Figure 1. Forty-four hours post-transfection, the cells were treated with 30 μM MG132 for 4 h, and fractionated into cytoplasmic and nuclear extracts as described in Materials and Methods. Anti-FLAG immunoprecipitation or anti HA-ubiquitin immunoprecipitation was performed to pull down HPV16 E2 or HA-ubiquitin conjugated proteins from both cytoplasmic and nuclear extracts. The purified immune complexes were analyzed by Western blot (Figure 1). For the FLAG immunoprecipitation, despite the fact that the majority of E2 is present in the nucleus (Figure 1A, FLAG blot, compare lane 3 and 5 to lane 4 and 6, respectively), only a small amount of E2 ubiquitin conjugates were isolated from the nuclear extract (Figure 1A, HA blot, lane 12). The vast majority of the E2 ubiquitin conjugates were isolated from the cytoplasmic extract (Figure 1A, HA blot, compare lane 11 to 12). This is further supported by the ubiquitin Western blot that detects E2 conjugated with both endogenous and HA-tagged ubiquitin (Figure 1A, Ub blot, compare lane 11 to 12). For the HA-ubiquitin immunoprecipitation, the majority of E2 is also present in the nucleus (Figure 1B, FLAG blot, compare lane 1 and 5 to lane 2 and 6, respectively). Similar to anti FLAG immunoprecipitation, most of the ubiquitin conjugates were isolated from the cytoplasmic extract (Figure 1B, HA blot and ubiquitin blot, compare lane 9 and 11 to lane 10 and 12, respectively). After HA-ubiquitin immunoprecipitation, E2 was detected in both the nuclear and cytoplasmic extracts (Figure 1B, FLAG blot, lane 11 and 12); however, a dark smear of high molecular weight E2 ladder was seen in the cytoplasmic fraction, indicating that E2 is more efficiently polyubiquitylated in this compartment. These results demonstrate that E2 is mainly ubiquitylated in the cytoplasm. 

#### 2.1.2. Brd4 Enhances E2 Nuclear Retention

**Figure 2:**
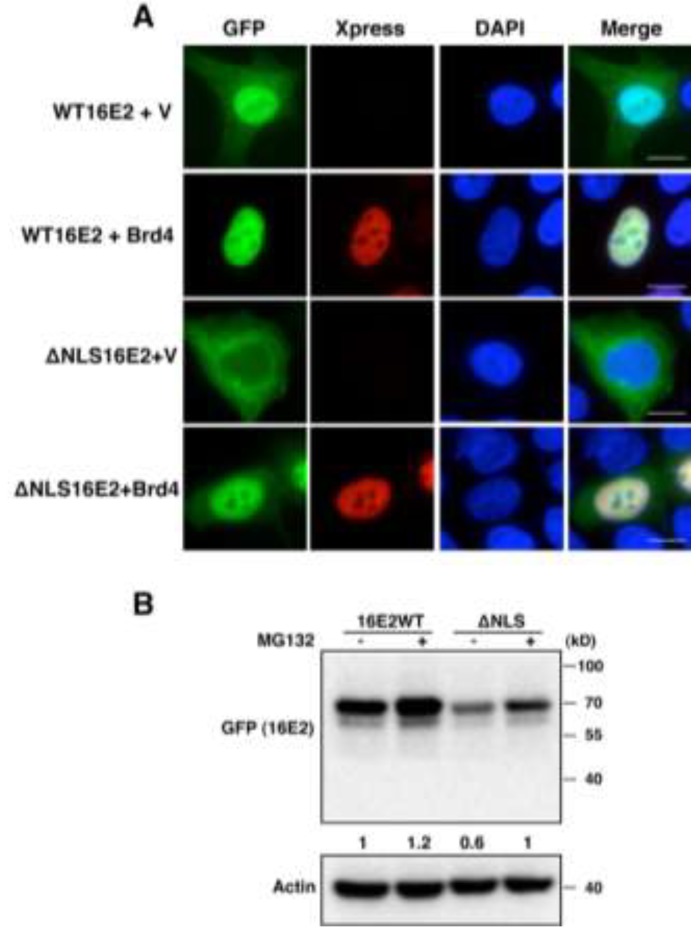
Full-length Brd4 recruits HPV16 E2 and a HPV16 E2 nuclear localization mutant to the nucleus and prevents their degradation by the proteasome. (**A**) HEK293T cells were transfected with either an empty pcDNA4C vector (V) or pcDNA4C-hBrd4 and a GFP construct encoding wild type HPV16 E2 (WT16E2) or ΔNLS-HPV16 E2 mutant (ΔNLS16E2). Transfected cells were stained with Xpress antibody (red) for Brd4 and counterstained with DAPI. Bar, 5 μM; (**B**) HEK293T cells were transfected with either pEGFP-C1-HPV16 E2 (16E2WT) or pEGFP-C1-ΔNLS16 E2 (ΔNLS). Forty-four hours post-transfection, the cells were treated with either 30 μM MG132 or DMSO for 4 h, and lysed as described in Materials and Methods. A portion (30 μg) of total cell lysates was resolved on a SDS-PAGE gel and immunoblotted with antibodies recognizing GFP (for HPV16 E2s) and actin. This experiment was repeated three times with similar results. Protein bands from the immunoblots were quantitated with Image J software, using the actin signal for normalization. Signal intensities were then normalized to 16E2WT treated with dimethyl sulfoxide (DMSO) (lane 1).

It has been previously demonstrated that direct protein interaction between Brd4 and E2 is essential for increasing the steady-state level and stability of E2 proteins [9,25]. Furthermore, Brd4 overexpression has been shown to increase the stability of E2 proteins fused to *Renilla* luciferase [26]. As we find that E2 is mainly ubiquitylated in the host cytoplasm (Figure 1), we speculated that Brd4 might cause E2 stabilization by retaining E2 inside the nucleus and shielding it from the cytoplasmic ubiquitylation machinery, thereby preventing its degradation by the proteasome. We, therefore, examined how the interaction of these two proteins affects the subcellular localization of E2. HEK293T cells were transfected with a GFP-HPV16E2 expression construct together with either an empty vector or a construct encoding Brd4 fused to an Xpress tag. The cells were subjected to Xpress immunofluorescent staining. As shown in Figure 2A, in the absence of Xpress-Brd4, GFP-tagged HPV16 E2 localizes predominantly to the nucleus. However, some faint E2 signal is also present in the cytoplasm (Figure 2A, WT16E2 + V panel). This is consistent with our fractionation experiments (Figure 1) and a previous study reporting that high-risk HPV E2 proteins are present in both the nucleus and the cytoplasm due to exportin-1 receptor (CRM1)-dependent nucleo-cytoplasmic shuttling [27]. In Xpress-Brd4 expressing cells, Xpress-Brd4 localizes exclusively to the nucleus (Figure 2A, WT16E2 + Brd4 panel, Xpress). Interestingly, the GFP-HPV16 E2 co-expressed with the Brd4 also becomes strictly nuclear (Figure 2A, WT16E2 + Brd4 panel, GFP). When compared to the cells without the Xpress-Brd4 expression, E2 signal is enhanced in the presence of Xpress-Brd4 expression (Figure 2A, compare GFP signals in WT16E2 + Brd4 panel with WT16E2 + V panel), supporting a role of Xpress-Brd4 in stabilizing E2 through recruiting and retaining it in the nucleus. 

To more directly test that Brd4 can retain E2 in the nucleus, we examined an E2 mutant that is deficient in nuclear localization. The nuclear localization signal (NLS) of high-risk HPV16 E2 protein has been well characterized [28]. Deletion analysis of GFP-HPV16 E2 has identified an alpha helix NLS within the DNA-binding region that is essential for nuclear localization of HPV16 E2 [28]. We, therefore, generated a nuclear-localization-defective mutant by deleting the HPV16 E2 NLS (GFP-ΔNLS16E2, see Materials and Methods). Brd4 immunoprecipitation shows that both wild type 16E2 and ΔNLS16E2 retain binding activity with Brd4 (Supplementary Figure S1), suggesting that ΔNLS16E2 is properly folded. As predicted, the GFP-ΔNLS16E2 mutant is largely excluded from the nucleus. Moreover, the mutant protein is detected at a much lower level than the wild type HPV16 E2 (Figure 2A, compare ΔNLS16E2 + V panel to WT16E2 + V panel). This lower steady-state level of E2 was also confirmed by a more quantitative Western blotting analysis (Figure 2B). These results suggest that blocking E2 nuclear localization can lead to E2 destabilization. We then tested if expression of Xpress-Brd4 molecules could also lead to the nuclear retention and stabilization of these E2 mutants. Remarkably, when co-expressed with Xpress-Brd4, the ΔNLS16E2 signal becomes highly enriched inside the nucleus (Figure 2A, compare ΔNLS16E2 + Brd4 panel with ΔNLS16E2 + V panel). This Brd4 recruitment and stabilization of E2 was further validated in a sub-cellular fraction assay, in which Brd4 recruits both wild type 16E2 and ΔNLS16E2 into the nucleus (Supplementary Figure S2, compare lanes 3 and 7 to lanes 4 and 8, respectively).

To determine if the ΔNLS16E2 is more sensitive to proteasomal degradation, we expressed both wild type and nuclear-localization-defective mutant HPV16 E2 proteins in HEK293T cells and compared their levels in the presence and absence of the proteasome inhibitor, MG132. Western blotting analysis shows that ΔNLS16E2 is present at a much lower steady-state level than the wild type HPV16 E2 in cells without MG132 treatment (Figure 2B, GFP blot). However, treatment of these cells with MG132 can significantly increase the ΔNLS16E2 level (Figure 2B). These results show that the nuclear-localization-defective E2 mutants are present at low steady-state levels and are rapidly degraded by the ubiquitin-proteasome pathway in cells. 

**Figure 3:**
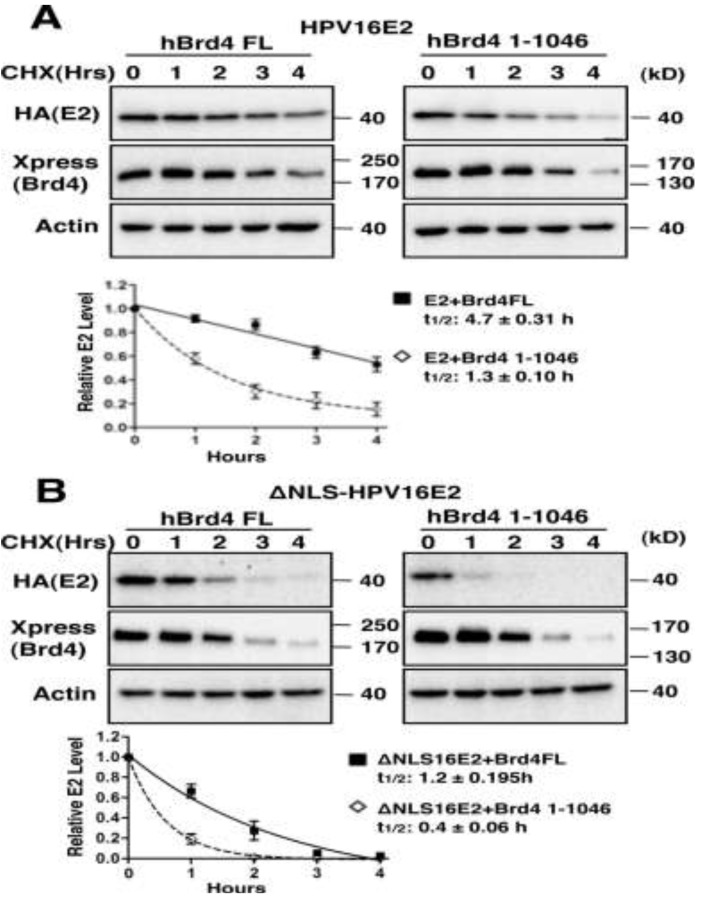
Brd4 stabilizes both HPV16 E2 and an HPV16 E2 nuclear localization mutant. (**A**) C33A cells were transfected with pOZN-HPV16E2 and either pcDNA4C-hBrd4FL or pcDNA4C-hBrd4 1-1046. Cells were treated with 100 μg/mL cycloheximide (CHX) at 24 h after transfection for the lengths of time indicated. Cell lysates were harvested and analyzed by Western blotting using HA (for 16E2), Xpress (for Brd4) and actin antibodies. Representative blots are shown. Protein bands from the immunoblots were quantitated with Image J software, using the actin signal for normalization. Signal intensities were then normalized to the 0 h time point. Error bars represent the standard deviation from three independent experiments. Half-lives were calculated using a one-phase exponential decay model; (**B**) C33A cells were transfected with pOZN-ΔNLSHPV16E2 and, either, pcDNA4C-hBrd4FL or pcDNA4C-hBrd4 1-1046. Cells were treated and analyzed as in (**A**).

#### 2.1.3. Brd4-Mediated E2 Nuclear Retention is Essential for E2 Stabilization

Co-expression of Brd4 with GFP-tagged 16E2 or ΔNLS16E2 leads to significantly elevated fluorescence signal for both proteins in the nucleus (Figure 2A), as well as a dramatic increase in nuclear E2 Western blot signal in fractionation experiments (Supplementary Figure S2). To determine if the enhanced E2 protein level induced by Brd4 expression is due to E2 stabilization, we performed a cycloheximide blocking/chasing analysis to compare E2 protein stability in the presence of full length Brd4 or the Brd4 1-1046 mutant that lacks the Brd4 CTD for E2 binding [11,14]. C33A cells (a papillomavirus-negative cervical cancer cell line) were co-transfected with either HA-tagged wild type 16E2 or the ΔNLS16E2 expression construct and an Xpress-tagged construct encoding either full-length Brd4 (hBrd4 FL) or the Brd4 1-1046 mutant. Twenty-four hours post-transfection, cells were treated with cycloheximide to inhibit protein synthesis and the protein stability was analyzed by Western blotting (Figure 3A,B). The half-life of HPV16E2 increases from 1.3 h when co-expressed with hBrd4 1-1046 to 4.7 h when co-expressed with hBrd4FL, confirming that Brd4 binding is required for E2 stabilization. A student’s T-test shows this difference is statistically significant (*p <* 0.05). As expected, ΔNLS16E2 shows a significantly lower half-life (0.4 h) in the presence of Brd4 1-1046 and the half-life increases to 1.2 h when co-expressed with hBrd4 FL. In summary, when compared to Brd4 1-1046, co-expression of full-length Brd4 leads to a significant increase (student’s *t*-test, *p <* 0.05) in the stability of both wild type 16E2 and ΔNLS16E2 (Figure 3A,B). Deletion of the Brd4 CTD in the Brd4 1-1046 abrogates E2 binding and allows for a more rapid degradation of both wild type 16E2 and ΔNLS16E2 (Figure 3A,B). This result suggests that Brd4 can stabilize both wild type 16E2 and ΔNLS16E2 possibly by tethering them within the nucleus to prevent E2 degradation. It is clear that wild type 16E2 is consistently more stable than ΔNLS16E2 when co-expressed with either hBrd4 FL or Brd4 1-1046, indicating that the nuclear localization function of wild type 16E2 may contribute to its stability. 

#### 2.1.4. Interaction with Brd4 is Responsible for E2 Stabilization

Brd4 mutants lacking the CTD had an attenuated ability to stabilize HPV 16 E2 proteins. To test whether this phenomenon was a more general property of papillomavirus E2 proteins and that direct binding to Brd4 was required for this stabilization, we tested for retention and stabilization of the Bovine Papillomavirus Type 1 (BPV1) proteins E2TA and E2TR (a truncated E2TA lacking the transactivating region required for interaction with Brd4) [11]. C33A cells stably expressing either E2TA or E2TR were transfected with expression constructs encoding hBrd4 FL or Brd4 1-1046. Twenty-four hours post-transfection, cells were treated with cycloheximide to block protein synthesis. E2TA and E2TR proteins isolated at different time points after cycloheximide treatment were analyzed by Western blotting analysis (Supplementary Figure S3). The data show that co-expression of full-length Brd4 significantly increases the half-life of E2TA (from 2.0 h when Brd4 1-1046 is expressed to 5.8 h when full-length Brd4 is expressed, student’s *t*-test, *p <* 0.05). However, the half-life of E2TR remains nearly unchanged when either the full-length Brd4 or Brd4 1-1046 is expressed (Supplementary Figure S3, *t*-test, *p =* 0.904). This experiment suggests that direct protein-protein interaction between the N-terminal domain of E2TA and the C-terminal domain of Brd4 leads to the stabilization of E2 proteins in cells. 

**Figure 4:**
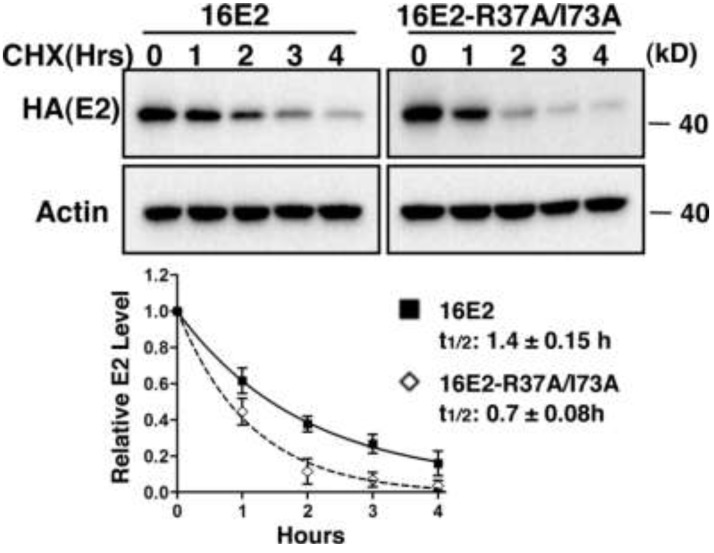
Disruption of E2 interaction with endogenous Brd4 decreases E2 protein stability. C33A cells were transfected with pOZN-16E2 or pOZN-16E2-R37A/I73A expression construct. Cells were treated with 100 μg/mL cycloheximide (CHX) at 24 h after transfection for the lengths of time indicated. Cell lysates were harvested and analyzed by Western blotting using HA (for 16E2) and actin antibodies. Representative blots are shown. Protein bands from the immunoblots were quantitated with Image J, using the actin signal for normalization. Relative E2 level and half-lives were calculated and presented as in Figure 3A. This experiment was repeated three times with similar results.

We, next, sought to confirm these findings with HPV16 E2 using the R37A/I73A mutant that is defective for Brd4 binding [19]. We also wished to confirm that this stabilization phenomenon could be seen with endogenously expressed Brd4 protein. C33A cells were transfected with wild type 16E2 or 16E2-R37A/I73A expression constructs and the half-lives of both proteins were analyzed by cycloheximide blocking/chasing analysis. As shown in Figure 4, the stability of 16E2-R37A/I73A (half-life, 0.7 h) is significantly reduced (student’s *t*-test, *p <* 0.05) compared to wild type 16E2 (half-life, 1.4 h). These experiments demonstrate that preventing E2 binding to endogenous Brd4 leads to its destabilization, suggesting that interaction with endogenous Brd4 is important for maintaining E2 stability. 

#### 2.1.5. Tethering E2 Proteins to Host Chromatin Increases E2 Stability

**Figure 5:**
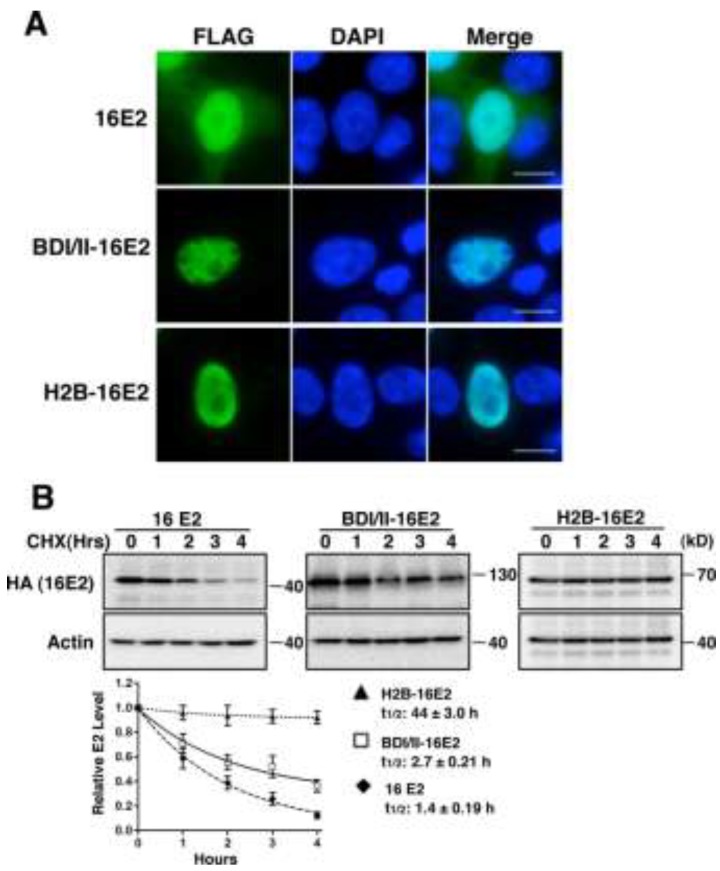
Tethering of HPV16 E2 to host chromatin increases E2 stability. (**A**) 293T cells were transfected with pOZN-16E2, pOZN-BDI/II-16E2, or pOZN-H2B-16E2. Transfected cells were stained with FLAG antibody (green) for 16E2 and counterstained with DAPI. Bar, 5 μM; (**B**) HEK293T cells were transfected with pOZN-16E2, pOZN-BDI/II-16E2 or pOZN-H2B-16E2. Cells were treated with 100 μg/mL cycloheximide (CHX) at 24 h after transfection for the lengths of time indicated. Cell lysates were harvested and analyzed by Western blotting using HA (16E2) and actin antibodies. Representative blots are shown. Protein bands from the immunoblots were quantitated with Image J, using the actin signal for normalization. Relative E2 level and half-lives were calculated and presented, as in Figure 3A. This experiment was performed three times with similar results.

To further prove that chromatin association via Brd4 can lead to E2 stabilization, we fused either the double bromodomains of Brd4 (BDI/II) or histone H2B to the N-terminus of the 16E2 protein. The BDI/II domain can bind to acetylated histone on chromatin whereas H2B can be incorporated into chromatin directly. To confirm that BDI/II and H2B can tether E2 to chromatin, immunofluorescent staining was performed to detect the sub-cellular localization of wild type 16E2, BDI/II-16E2, and H2B-16E2 expressed in 293Tcells. As shown in Figure 5A, the majority of wild type 16E2 is present in the nucleus, but some small amount of E2 protein also localizes to the cytoplasm. Compared with wild type 16E2, the BDI/II-16E2 and H2B-16E2 show a chromatin-like staining (Figure 5A). 

To test for protein stability, HEK293T cells were transfected with recombinant constructs encoding wild type 16E2, BDI/II-16E2, or H2B-16E2. Cycloheximide blocking/chasing analysis was performed to detect protein stability. As shown in Figure 5B, both BDI/II-16E2 and H2B-16E2 show dramatically increased half-lives compared to the wild type E2 (One way ANOVA, *p <* 0.05). Additional studies show that when BPV1 E2TA or HPV11 E2 were fused to BDI/II or H2B, their half lives are also increased (Supplementary Figure S4). Sub-cellular fraction analysis also shows BDI/II and H2B localize 16E2 to the nucleus (Supplementary Figure S5). Together, these studies demonstrate that tethering E2 proteins to chromatin can increase their stability. These results provide evidence to support the hypothesis that Brd4 mediated E2 chromatin association contributes to E2 stabilization. 

### 2.2. Discussion

Brd4 functions as a molecular adapter that bridges the interaction between viral E2 proteins and host chromatin. Studies of E2 and Brd4 have established Brd4 as a key host receptor that regulates the multiple functions of E2. Brd4 has also been implicated in modulating E2 stability. In this study, we demonstrate that Brd4 binding significantly enhances E2 nuclear retention and contributes to its stabilization. We show that Brd4 is able to redirect a nuclear-localization-defective E2 mutant back into the nucleus. Normally, the nuclear-localization-defective mutant is rapidly degraded by the ubiquitin-proteasome pathway in the cytoplasm. However, the Brd4-induced nuclear retention of this E2 mutant significantly increases its stability, establishing nuclear retention as one of the mechanisms for E2 stabilization. In view of the fact that E2 is mostly ubiquitylated in the cytoplasm, our study supports a model in which the Brd4-mediated nuclear retention of E2 protein prevents its ubiquitylation and degradation in the cytoplasm, contributing to its stabilization in the nucleus. Our data provide an additional mechanism by which chromatin-associated Brd4 regulates E2 functions.

We also directly tested how inhibition of E2 binding to endogenous Brd4 could affect E2 stability. Knockdown of endogenous Brd4 arrests cells in G1 phase [29,30], and E2 stabilization has been linked to S-phase progression [31]. Therefore, knockdown of Brd4 was not a viable strategy for probing endogenous Brd4’s role in E2 stability. To avoid this complication, we tested the stability of the 16E2-R37A/I73A mutant that is defective in Brd4 binding and found that it was significantly reduced when compared to wild type 16E2 (Figure 4). We saw similar results with BPV1 E2TA and its E2TR isoform, which does not bind Brd4 (Supplementary Figure S3). These studies confirm that the ability to interact with Brd4 is important for maintaining E2 stability.

E2 ubiquitylation occurs predominantly in the cytoplasm (Figure 1), suggesting that the major E2 ubiquitylation machinery may be present in the cytoplasm. By retaining E2 inside the nucleus, Brd4 proteins may physically block the E2 interaction with its E3 ubiquitin ligases, thus, inhibiting its ubiquitylation and degradation by the proteasome. This notion is in line with a study showing that the Brd4 CTD can block the E2 interaction with the Cullin-3 ubiquitin protein ligase to inhibit E2 ubiquitylation [9]. In addition to Brd4, other cellular proteins have also been implicated in the regulation of E2 stability. For instance, Tax1BP1, an essential component of an A20 ubiquitin-editing complex, regulates the steady-state level of E2 by preventing its proteasomal degradation [32]. Another study has reported that endogenous TAX1BP1 localizes to intranuclear speckles [33]. It will be interesting to test if this nuclear protein also stabilizes E2 through a nuclear retention mechanism. 

Throughout our study, we observed that Xpress-Brd4 has a stronger effect in recruiting and stabilizing HPV16 E2 inside the nucleus than it does for BPV1 E2TA. This could be due to the fact that E2TA binds to endogenous Brd4 with a much higher affinity than HPV16 E2 ([23] and data not shown). The Xpress-Brd4 molecules are, therefore, unable to influence E2TA as dramatically as they are for HPV16 E2. The stronger E2TA-Brd4 interaction may also explain why E2TA is more stable than HPV16 E2 and is less affected by tethering to chromatin (Figure 5 and Supplementary Figure S4 ).

A previous study and our current work show that E2 shuttles between the cytoplasm and the nucleus [27]. On the other hand, Brd4 remains mostly nuclear through interaction with acetylated histones [13]. This phenomenon suggests that E2 may be constantly binding to and releasing from Brd4. To mimic the Brd4-mediated tethering of E2 proteins to host chromatin, we fused E2 proteins to the Brd4 BDI/II and H2B. Not surprisingly, both the BDI/II and H2B fusions lead to significant stabilization of E2 proteins. Among these fusion proteins, BDI/II-E2s interact with chromatin in a manner that most closely resembles the Brd4-mediated E2 and chromatin interaction. Presumably, because the BDI/II can also bind to acetylated histone with a rapid “ON/OFF” mode [13], it leads to a less dramatic stabilization of BDI/II-E2 fusions than H2B, which is more stably integrated into chromatin. These studies therefore provide additional evidence to support that the Brd4-mediated E2 chromatin association can contribute to its stabilization inside the nucleus. Although the studies of BDI/II- or H2B-fused E2s indicate that direct incorporation into chromatin does stabilize E2 proteins, it is possible, however, that these 16E2 fusion proteins still interact with Brd4 on the chromatin. From our data, we cannot rule out the possibility that Brd4’s interaction with E2 stabilizes it using mechanisms beyond chromatin tethering. 

It has been suggested that Brd4 may stabilize the chromatin-associated E2 at the basal layer of HPV-infected epithelium to maintain E2 at sufficient level for viral activities such as DNA replication [10]. E2 stabilization by Brd4 was most robustly seen when Brd4 was ectopically overexpressed (compare differences in half-lives between Figure 3 and Figure 4). We hypothesize that exogenously expressed E2 saturates endogenous Brd4, which can only stabilize a small pool of ectopically expressed E2. Overexpression of Brd4 allows for a more robust E2 stabilization phenotype. Although ectopic expression of Brd4 may be seen as an artificial environment to test for E2 stability, it is well established that E2 expression is generally low within the basal epithelial layer and would not likely saturate endogenous Brd4 levels [34]. On the other hand, by immunofluorescent staining of Brd4 in an organotypic raft culture established from immortalized human foreskin keratinocytes, we observed that Brd4 is highly expressed in the basal epithelial layer and that the Brd4 level further increases during keratinocyte differentiation (data not shown). In normal HPV infections, there is likely a lot more Brd4 than E2 in the infected basal epithelial cells, which could lead to efficient retention and stabilization of E2 in the nucleus. Our expression system may also reflect a late point in viral infection, at which stage the increase in Brd4 levels upon cellular differentiation may further increase the E2 level to promote viral genome replication and transcription. 

As Brd4 functions in tethering the viral episomes to mitotic chromosomes and in regulating both E2-mediated viral oncogene transactivation and repression, the high level of Brd4 expression in the basal epithelial layer suggests that Brd4 could promote E2 functions at the place where the virus first infects host epithelium. How Brd4 regulates E2 stability during the productive viral life cycle in HPV-infected cells remains an important question for future studies. The involvement of the E2-Brd4 complex in multiple aspects of the papillomavirus life cycle establishes it as an important target for the development of antiviral therapeutics [11,14]. New insights into the mechanism of E2 stabilization during the viral life cycle will offer effective strategies to prevent papillomavirus-induced human cancer. 

## 3. Experimental Section

### 3.1. Cell Culture, Cell Lines, and Transfection

C33A and HEK293T cells were maintained in monolayer cultures in Dulbecco’s modified Eagle’s medium (Invitrogen, Carlsbad, CA, USA) containing 10% fetal calf serum (Hyclone, Logan, UT, USA). Cells were transfected with FuGENE 6 transfection reagent (Promega, Madison, WI, USA), Lipofectamine 2000 reagent (Invitrogen), or the calcium phosphate method as previously described [23]. 

### 3.2. Recombinant Plasmid Construction

Plasmids pOZN-E2TA, pOZN-E2TR, pcDNA3-HA-Ub, pCMV-FLAG-HPV16 E2, and pcDNA4C constructs encoding Xpress tagged hBrd4FL, and hBrd4 aa1-1046 have been described previously [9,11,20]. For pOZN-16E2, pOZN-ΔNLS16E2, and pOZN-16E2R37A/I73A constructs, DNA fragments encoding the full-length HPV16 E2, the E2 sequences with deleted NLS (aa298-308), and R37A/I73A mutations were cloned individually into pOZN using XhoI and NotI sites. For pOZN-BDI/II-16E2 and pOZN-BDI/II-E2TA, the DNA sequence encoding aa 1-470 of hBrd4 was cloned into pOZN-16E2 and pOZN-E2TA using the Xho I site. For pOZN-H2B-16E2, pOZN-H2B-E2TA, and pOZN-H2B-11E2, the DNA sequence encoding H2B was cloned into pOZN-16E2, pOZN-E2TA, and pOZN-11E2 respectively using the Xho I site. For pEGFP-C1-HPV16 E2 and pEGFP-C1-ΔNLS16E2 plasmids, DNA fragments spanning either the full-length HPV16 E2 or the E2 sequence with deleted NLS (aa298-308) were cloned into pEGFP-C1 using the BglII and EcoRI sites. All constructs were verified by DNA sequencing.

### 3.3. Protein Half-Life Cycloheximide Blocking/Chasing Analysis

HEK293T or C33A growing in 6 cm dishes were transfected with the indicated plasmids. At 24 h post-transfection, cells were treated with 100 μg/mL cycloheximide to block protein synthesis. Cells were harvested at 0, 1, 2, 3, or 4 h after treatment and analyzed by Western blotting. Protein bands from the immunoblots were quantitated with Image J, using the actin signal for normalization. The half-lives of E2 proteins were calculated using a one-phase exponential-decay model. 

### 3.4. Western Blotting

For protein analysis, cells were harvested at the indicated times after transfection. In most cases, cells were lysed in extraction buffer (10 mM HEPES pH 7.9, 300 mM NaCl, 3 mM MgCl_2_, 1 mM DTT, and 1 mM PMSF, supplemented with protease inhibitor cocktails (Roche, Indianapolis, IN, USA) and Ser/Thr protein phosphatase inhibitor cocktails (Sigma, St. Louis, MO, USA) and passed through 25-gauge needles 10 times. After 20 min incubation on ice, lysates were clarified by centrifugation at 5,000 rpm for 5 min. For the cycloheximide blocking/chasing analysis to detect the stability of E2 proteins and E2 tagged with BDI/II or H2B, cells were lysed in SDS-PAGE sample buffer containing 50 mM Tris, pH 6.8, 2% SDS, 10% glycerol, 5% 2-mercaptoethanol, 3.5 mM sodium butyrate, 2.5 mM TSA, and 1 mM phenylmethylsulfonyl fluoride (PMSF), supplemented with protease inhibitor cocktails (Roche) and Ser/Thr protein phosphatase inhibitor cocktails (Sigma). Cells were sonicated at 50 W with three 30-second pulses. Extracts (20 μg) were resolved on a SDS-PAGE gel. Proteins were transferred to a polyvinylidene fluoride (PVDF) membrane (PerkinElmer, Waltham, MA, USA) and blotted with Xpress antibody (Invitrogen), anti-HA HRP antibody (Roche), GFP antibody (Santa Cruz, Dallas, TX, USA), actin antibody (Chemicon, Billerica, MA, USA), Ub antibody (Biomol, Farmingdale, NY, USA), FLAG M2 antibody (Sigma), FLAG-HRP (Sigma), α-tubulin antibody (Sigma), SP1 antibody (Santa Cruz), and an antibody recognizing aa156-284 of Brd4. 

### 3.5. Immunoprecipitation

For anti-FLAG HPV16 E2 immunoprecipitation, HEK293T cells were transfected with plasmids encoding HA-tagged ubiquitin, FLAG-tagged HPV16 E2 or both using the calcium phosphate method. Forty-four hours post-transfection, the cells were treated with 30 μM MG132 for 4 h. The cytoplasmic and nuclear extracts were prepared as described previously [35]. Briefly, the cells were lysed in buffer A (10 mM HEPES pH7.9, 10 mM KCl, 0.1 mM EDTA, 0.1 mM EGTA, and 1 mM DTT, supplemented with protease inhibitors (Roche)). The cells were incubated on ice for 10 min and NP-40 was added to a final concentration of 0.6%. After vortexing and centrifugation at 2,700 × g for 5 min, the supernatant was collected as cytoplasmic extract, and the nuclear pellet was resuspended in ice-cold buffer B (20 mM HEPES pH7.9, 0.4 M NaCl, 1 mM EDTA, 1 mM EGTA, and 1 mM DTT, supplemented with protease inhibitors). To extract the nuclear proteins, the nuclei were passed through a 21-gauge needle 5 times and extracted at 4 °C for 1 h. The nuclear proteins were isolated by centrifugation at 20,800 × g for 15 min. Cytoplasmic extract or nuclear extract was mixed with anti-FLAG M2 agarose beads (Sigma, pre-blocked with 1% BSA in PBS for 1 h at 4 °C) and rotated at 4 °C for 3.5 h. The beads were washed three times with 0.15 M KCl Base Buffer (0.15 M KCl, 20 mM Tris-Cl pH8.0, 10% Glycerol, 5 mM MgCl_2_, 0.1% Tween-20, 0.5 mM DTT and 0.2 mM PMSF) before elution with 30 μL SDS sample buffer. For HA-Ubiquitin immunoprecipitation, the cytoplasmic extract or nuclear extract was pre-cleared with normal mouse IgG and protein A beads (GE healthcare, Mickleton, NJ, USA) and rotated at 4 °C for 1 h. The extract was then mixed with HA antibody and rotated at 4 °C for another hour. The immune complexes were mixed with Protein A beads at 4 °C overnight. The beads were washed three times with 0.15 M KCl Base Buffer before elution with 30 μL SDS sample buffer. Aliquots (8 μL) of the immunoprecipitated proteins were resolved on an 8% SDS-PAGE gel and analyzed by Western blotting. 

### 3.6. Immunofluorescent Staining

Cells grown on coverslips were transfected with plasmids as indicated in the figure legends. Twenty-four hours post-transfection, cells were fixed with 3% paraformaldehyde in PBS. Cells were incubated in blocking/permeabilization buffer (0.5% Triton X-100 and 3% BSA in PBS) for 20 min at room temperature, and stained with specific primary antibodies (as described in the legends) at room temperature for 60 min. After incubation, cells were washed 3 times with blocking/permeabilization buffer and incubated with Alexa Fluor 594 or 488 goat anti-mouse IgG (Molecular Probes, Grand Island, NY, USA) for an additional 60 min. After incubation with the secondary antibodies, cells were counterstained with DAPI (4',6'-diamidino-2-phenylindole) and examined with an Olympus IX81 inverted fluorescence microscope.

### 3.7. Statistical Analysis

Statistical analysis was performed using T-test or one-way ANOVA with the Graphpad Prism software (Version 5.0). *p <* 0.05 was considered statistically significant.

## 4. Conclusions

Papillomavirus E2 protein normally shuttles between the cytoplasm and the nucleus. E2 is mostly degraded by the ubiquitin-proteasome pathway in the cytoplasm. In this study, we demonstrate that the interaction with Brd4 recruits papillomavirus E2 proteins in the nucleus and contributes to their stabilization. Co-expression of Brd4 redirects the nuclear-localization-defective mutant into the nucleus and significantly increases its stability. We demonstrate that the HPV16 E2 mutant R37A/I73A which is deficient in binding Brd4 is less stable than wild type HPV16 E2 with endogenous levels of Brd4. We further demonstrate that tethering E2 proteins to chromatin as either double-bromodomain fusion proteins or histone H2B fusion proteins significantly stabilizes the E2 proteins. These studies reveal new mechanisms of Brd4-mediated E2 stabilization. Taken together, our studies demonstrate that interaction with Brd4 contributes to E2 nuclear retention and stabilizes E2 in the nucleus. 

## Supplementary Files

Supplementary File 1 Supplementary Materials (PDF, 1767 KB)
