# Supplementary material for: Brd4-Mediated Nuclear Retention of the Papillomavirus E2 Protein Contributes to Its Stabilization in Host Cells
Source: Viruses. 2014 Jan 20;6(1):319–35. doi: 10.3390/v6010319 (PMC3917445; doi:10.3390/v6010319)

Supplementary Materials

Figure S1. 16E2 and ΔNLS16E2 interact with Brd4. 293T cells were transfected with pEGFPC1–16E2 or pEGFPC1-ΔNLS16E2 together with pcDNA4C or pcDNA4C-Brd4. At 48 h post-transfection, cells were harvested for Brd4 immunoprecipitation. The purified immune complexes were resolved on a SDS-PAGE gel. Western blotting was performed using antibodies recognizing Brd4, Xpress (for ectopic Brd4), 16E2, or GFP (for E2).

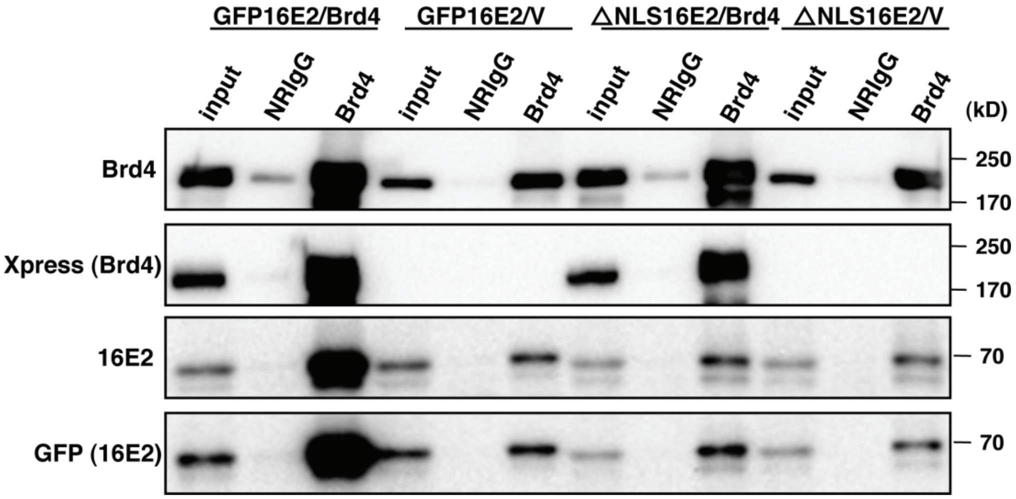

Figure S2. Brd4 recruits 16E2 and ΔNLS16E2 into nucleus and stabilizes them. 293T cells were transfected with pEGFPC1–16E2 or pEGFPC1-ΔNLS16E2 together with pcDNA4C or pcDNA4C-Brd4. At 48 h post-transfection, cells were lysed to obtain cytoplasmic extracts (C) and nuclear (N) extracts. The isolated cytoplasmic or nuclear extracts were analyzed by western blot with antibodies recognizing GFP (16E2), Xpress (ectopic Brd4), or Brd4. Brd4 was used as nuclear protein marker.

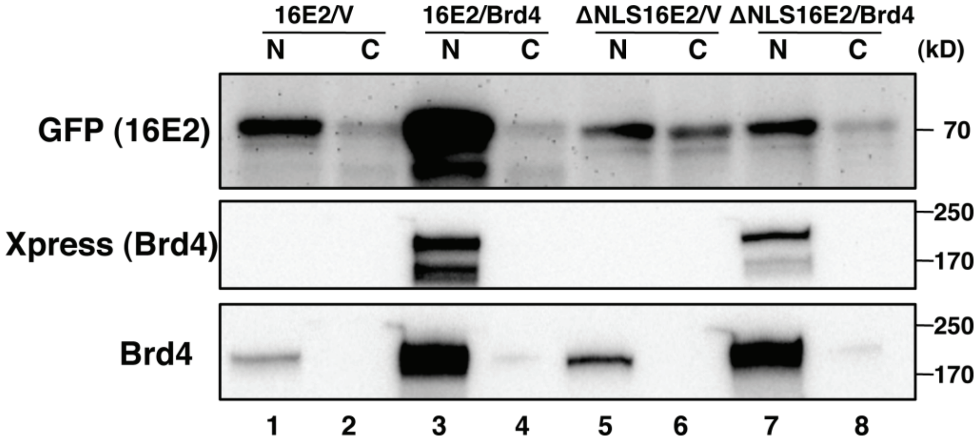

Figure S3. E2 proteins are stabilized via interaction with the C-terminal domain of Brd4. (**A**) C33A cells stably expressing HA-E2TA were transfected with pcDNA4C-hBrd4FL or pcDNA4C-hBrd4 1-1046, a truncation mutant that lacks the Brd4 CTD region required for E2 binding. Cells were treated with 100 μg/mL cycloheximide (CHX) at 24 h after transfection for the length of time indicated. Cell lysates were harvested and analyzed by Western blotting using anti-HA HRP, Xpress and actin antibodies. Protein bands from the immunoblots were quantitated with Image J software, using the actin signal for normalization. Representative blots are shown. Relative E2 level and half-lives were calculated and presented as in Figure 3A; (**B**) C33A cells stably expressing E2TR were transfected and analyzed as in (**A**).

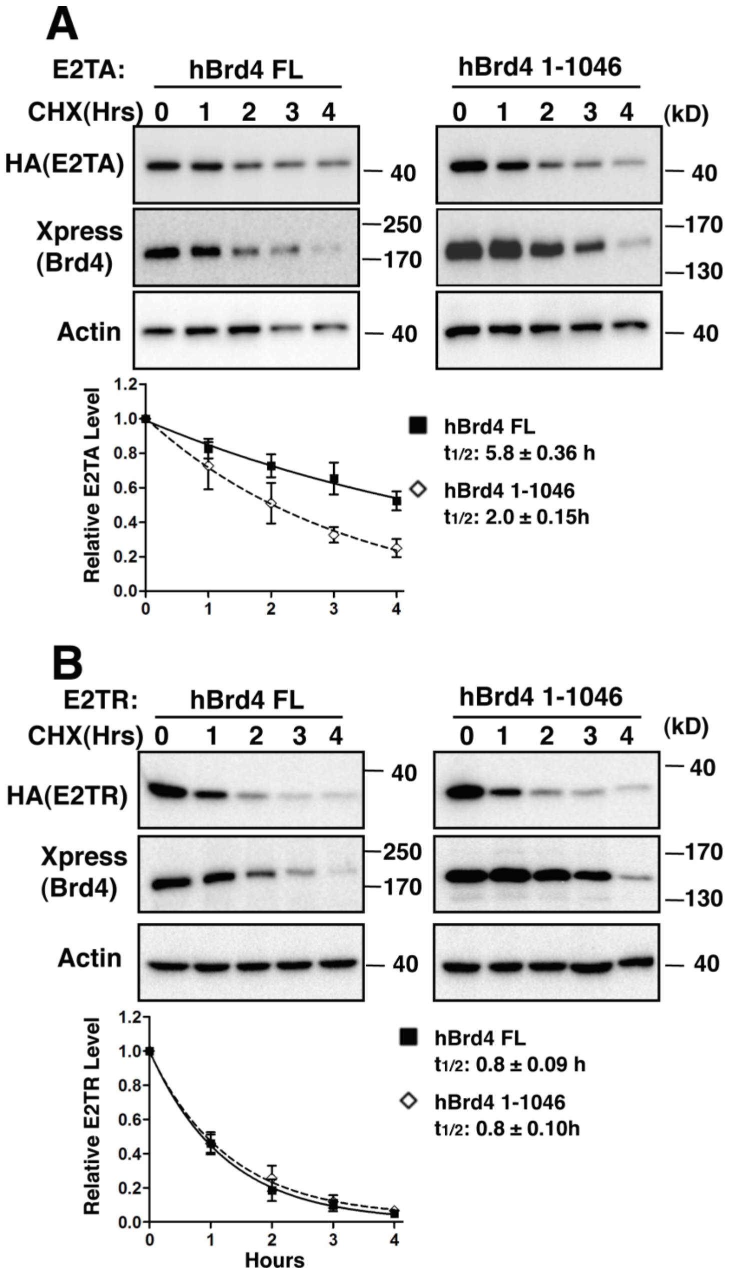

Figure S4. Chromatin association enhances the stability of BPV1 and HPV11 E2s. (**A**) HEK293T cells were transfected with pOZN-E2TA, pOZN-BDI/II-E2TA or pOZN-H2B-E2TA**.** Cells were treated with 100 µg/mL cycloheximide (CHX) at 24 h after transfection for the lengths of time indicated. Cell lysates were harvested and analyzed by Western blotting using anti-HA HRP and actin antibodies. Representative blots are shown. Protein bands from the immunoblots were quantitated with Image J, using the actin signal for normalization. Relative E2 level and half-lives were calculated and presented as in Figure 3A; (**B**) The stability of HPV11E2 and H2B-11E2 were analyzed as in (**A**).

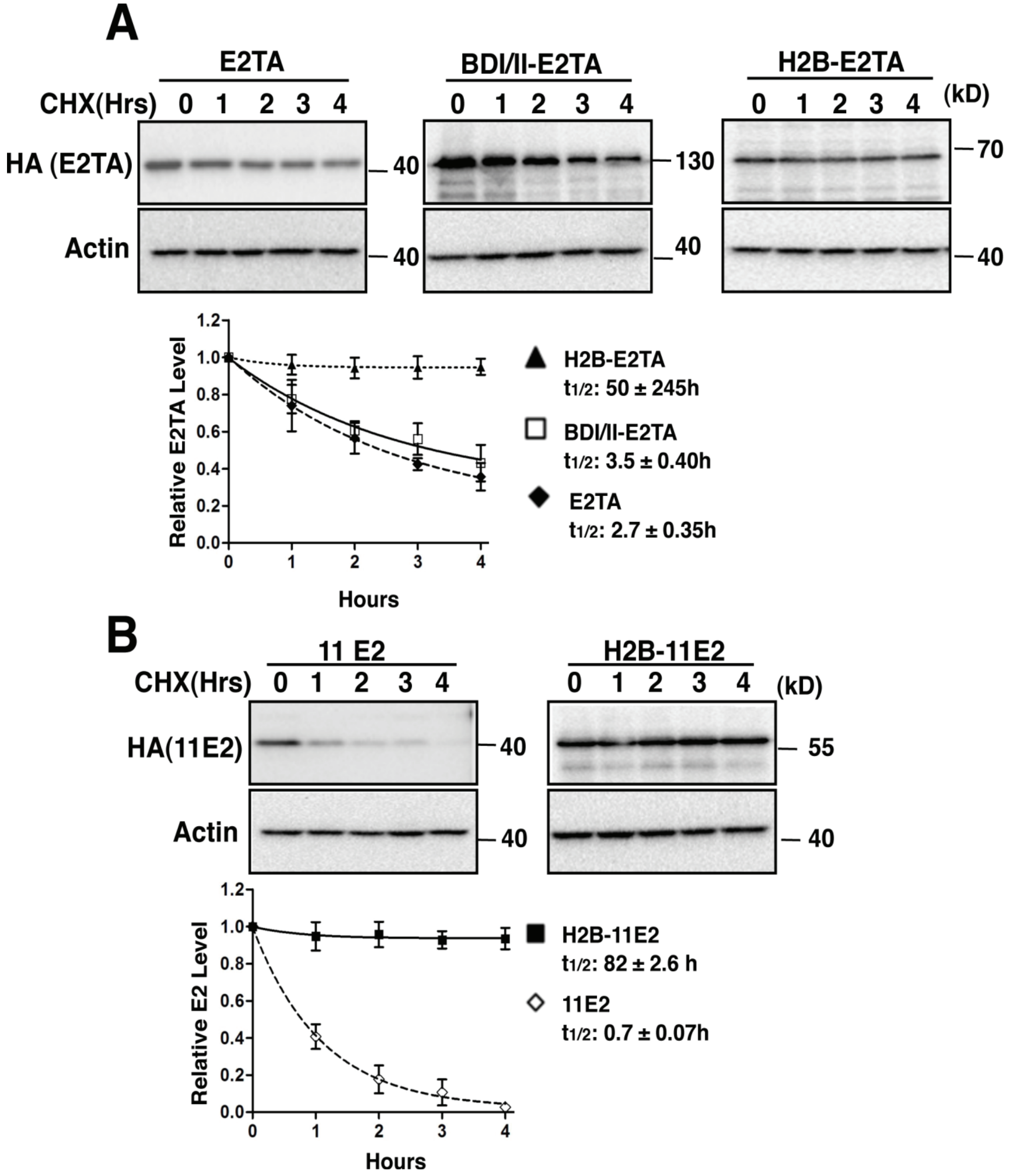

Figure S5. Tethering 16E2 to chromatin increases its stability. 293T cells were transfected with pOZN-16E2, pOZN-BDI/II-16E2, or pOZN-H2B-16E2. At 48 h post-transfection, cells were lysed to obtain cytoplasmic extracts (C) and nuclear extracts (N). The isolated cytoplasmic or nuclear extracts were analyzed by western blot with antibodies recognizing HA (16E2), Brd4 or α tubulin. Brd4 was used as nuclear protein marker and α tubulin as a cytoplasmic marker.

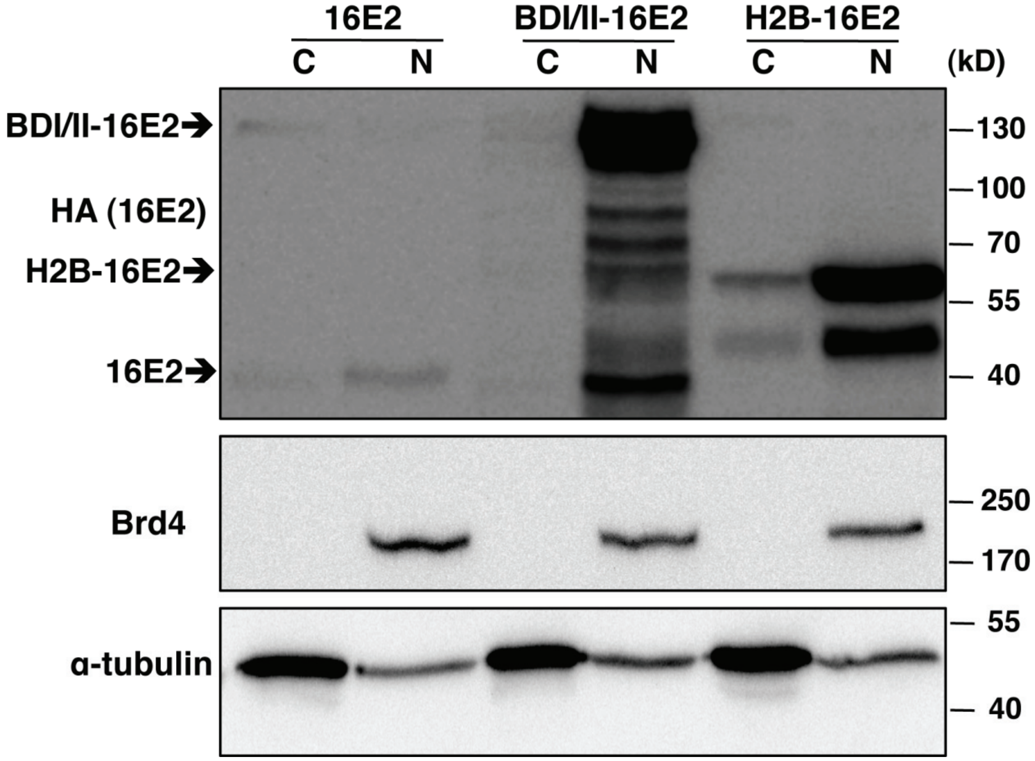

Supplement: Supplementary File 1 — Supplementary Materials (PDF, 1767 KB) [file viruses-06-00319-s001.pdf]
